# Mesenchymal stem cells genetically engineered to express platelet-derived growth factor and heme oxygenase-1 ameliorate osteoarthritis in a canine model

**DOI:** 10.1186/s13018-020-02178-4

**Published:** 2021-01-11

**Authors:** Jiwon Oh, Yeon Sung Son, Wan Hee Kim, Oh-Kyeong Kwon, Byung-Jae Kang

**Affiliations:** 1 Department of Veterinary Clinical Sciences, College of Veterinary Medicine and Research Institute for Veterinary Science, Seoul National University, Seoul, 08826 South Korea; 2 Medical Research Center, College of Medicine, Seoul National University, Seoul, 03080 South Korea; 3 BK21 PLUS Creative Veterinary Research Center, Seoul National University, Seoul, 08826 South Korea

**Keywords:** Mesenchymal stem cells, Osteoarthritis, Platelet-derived growth factor, Heme oxygenase-1

## Abstract

**Background:**

Mesenchymal stem cells (MSCs) are used for the treatment of osteoarthritis (OA), and MSC genetic engineering is expected to enhance cartilage repair. Here, we aimed to investigate the effect of MSCs overexpressing platelet-derived growth factor (PDGF) or heme oxygenase-1 (HO-1) in chondrocytes and synovial cells with an OA phenotype and assess the in vivo efficacy of intra-articular injections of these MSCs in canine OA models.

**Methods:**

Canine adipose-derived MSCs were transfected with canine PDGF (PDGF-MSCs) or HO-1 (HO-1-MSCs) using lentiviral vectors. Canine chondrocytes or synovial cells were stimulated with lipopolysaccharide (LPS) to mimic the inflammatory OA model and then co-cultured with MSCs, PDGF-MSCs, or HO-1-MSCs for 24 h and 72 h. The mRNA levels of pro-inflammatory, extracellular matrix-degradative/synthetic, or pain-related factors were measured after co-culture by real-time PCR. Furthermore, a surgery-induced canine OA model was established and the dogs were randomized into four groups: normal saline (*n* = 4), MSCs (*n* = 4), PDGF-MSCs (*n* = 4), and HO-1-MSCs (*n* = 4). The OA symptoms, radiographic OA severity, and serum matrix metallopeptidase (MMP)-13 levels were assessed before and 10 weeks after treatment, to evaluate the safety and efficacy of the modified MSCs.

**Results:**

PDGF or HO-1 overexpression significantly reduced the expression of pro-inflammatory factors, MMP-13, and nerve growth factor elicited by LPS and increased that of aggrecan and collagen type 2 in chondrocytes (*P* < 0.05). In addition, the expression of aggrecanases was significantly downregulated in synovial cells, whereas that of tissue inhibitor of metalloproteinases was upregulated (*P* < 0.05). Furthermore, the co-cultured MSCs highly expressed genes that contributed to the maintenance of joint homeostasis (*P* < 0.05). In vivo studies showed that OA symptoms improved after administration of all MSCs. Also, PDGF-MSCs significantly improved limb function and reduced pain (*P* < 0.05). The results of the radiographic assessment and serum MMP-13 levels did not vary significantly compared to those of the control.

**Conclusions:**

Genetically modifying PDGF and HO-1 in MSCs is an effective strategy for treating OA, suggesting that PDGF-MSCs can be novel therapeutic agents for improving OA symptoms.

**Supplementary Information:**

The online version contains supplementary material available at 10.1186/s13018-020-02178-4.

## Background

Osteoarthritis (OA) is a multifactorial disease with diverse pathophysiology that affects the articular cartilage, subchondral bone, and synovium [1]. OA affects mostly humans, dogs, and cats, in all of which it leads to a significant deterioration in the quality of life [2, 3]. Development of OA includes the initiation of inflammation and degradation of the extracellular matrix (ECM) of the articular cartilage, which leads to pain and gradual loss of limb function [4].

Owing to their pluripotent and anti-inflammatory properties, mesenchymal stem cells (MSCs) have been used for treating musculoskeletal diseases [5, 6]. However, recent studies have shown that although these cells disappear rapidly after administration, they can exert a chondroprotective effect via paracrine signaling [7]. To overcome this problem, tissue engineering using gene-edited MSCs has become a novel approach for the treatment of OA [8, 9]. Promising results have been reported using this method in equine MSCs with the gene encoding interleukin (IL)-1 receptor antagonist [10]. In addition, in a rabbit model of an osteochondral defect, delivery of fibroblast growth factor-2 (FGF-2), sex-determining region Y-box 9 (SOX-9), or insulin growth factor-1 (IGF-1) yielded promising results [9, 11, 12]. The encouraging results of these studies motivated us to identify more factors that can improve the therapeutic efficacy of MSCs after gene transfer.

As platelet-rich plasma (PRP) is a valuable option for OA treatment, we focused on platelet-derived growth factor (PDGF), a key factor in PRP believed to support tissue regeneration and anti-inflammatory properties [13]. We have previously reported that MSCs which were genetically engineered to overexpress PDGF improved cutaneous wound healing in canine skin wound healing models [14]. We expect that MSCs will exert strong paracrine effects within the OA joint if genetically modified with PDGF.

Several studies have shown that antioxidant activity was lower in OA human cartilage than in its healthy counterpart [15], accompanied by downregulation of heme oxygenase-1 (HO-1) by inflammatory cytokines. HO-1 is induced by oxidative stress and can inhibit apoptosis due to its antioxidant properties [16]. In human OA synovial cells, transduction with a lentiviral vector expressing HO-1 downregulated degradative and inflammatory cytokines [17]. Based on these reports, we hypothesized that the administration of HO-1 via MSCs may modulate inflammation in OA.

Hence, in this study, we aimed to investigate whether canine adipose-derived MSCs (AD-MSCs) engineered to overexpress PDGF or HO-1 exhibit biological activity and therapeutic efficacy in vitro and in vivo, and analyzed the expression of the pro-inflammatory, ECM-synthetic/degradative, and pain-related factors. We focused on these two biofactors because they affect the cross-talk of MSCs with OA chondrocytes or synovial cells.

## Methods

All experimental protocols regarding animals in this study were approved by the Institutional Animal Care and Use Committee (IACUC) of the Seoul National University (SNU-180530-3-1 and SNU-200528-3).

### Isolation of canine AD-MSCs, chondrocytes, and synovial cells

Subcutaneous fat, normal articular cartilage, and synovium were harvested from the femoral head of dogs undergoing femoral head and neck excision. Tissue sampling was performed after obtaining informed consent at the Seoul National University Veterinary Medical Teaching Hospital (SNU VMTH). Macroscopically normal tissue was obtained. Four skeletally mature dogs of small to medium breed aged 1–5 years and weighing 3–12 kg were used in the study. The samples were stored in Hartman’s solution. The harvested tissue was washed in Dulbecco’s phosphate-buffered saline (DPBS; Sigma).

The adipose tissue was cut into small pieces and digested with collagenase type I (1 mg/mL, Sigma) for 1 h at 37 °C. The digested fragments were filtered through a 40-μm cell strainer and the cells were pelleted via centrifugation at 220×*g* for 5 min at 4 °C. The cells were resuspended in Dulbecco’s modified Eagle’s medium (DMEM; Pan Biotech) supplemented with 10% fetal bovine serum (FBS, Gibco) and 1% penicillin-streptomycin and cultured in a 5% humidified CO_2_ incubator at 37 °C. The culture medium was changed every 2 days. Subculture was performed at 80% cell confluence.

Canine articular chondrocytes were isolated using an enzymatic dissociation procedure described previously [18]. Articular cartilage was finely minced and digested with collagenase type II for 1 h at 37 °C. The minced pieces of cartilage were passed through a 70-μm cell strainer. The dispersed chondrocytes were resuspended in DMEM and used for experiments at passage 1. Canine synovial cells were cultured from the synovium using a modified version of a published protocol [19]. The synovial tissue digested with collagenase type I was filtered through a 70-μm cell strainer. The next process was performed as described above, and passage 1 of canine synovial cells was used for experiments.

### Flow cytometry

To characterize canine AD-MSCs, MSC-specific markers were analyzed using flow cytometry with a FACS Calibur (BD Biosciences), and data were evaluated using the CellQuest 3.0.1 software (Becton-Dickinson). The cells were incubated with primary antibodies, phycoerythrin (PE)-conjugated antibody, and fluorescein-conjugated antibody. Briefly, the cells were detached with Accutase (Stem Cell Technologies), followed by the addition of FITC-labeled anti-rat CD29 (555005, BD Pharmingen), anti-human CD105 (ab53318, Abcam), anti-dog CD45 (YKIX716.13, BioRad), anti-dog CD34 (1H6, BioRad), anti-human CD9 (hCD9, sc13118, Santa Cruz Biotechnology), anti-mouse CD9 (mCD9, sc18869, Santa Cruz), and anti-human CD91 (550495, BD Pharmigen), and PE-labeled anti-dog CD44 (ab58754, Abcam), anti-human CD73 (ab106697, Abcam), and anti-dog CD90 (ab33694, Abcam). For the detection of pluripotency markers, OCT-4, SOX-2, and NANOG were measured. The cells were fixed and permeabilized using the eBioscience^TM^ Foxp3 staining buffer set (#00-5523-00, Invitrogen), blocked in DPBS containing 5% FBS, and then stained with primary unconjugated OCT-4 (ab19857, Abcam), SOX-2 (ab97959, Abcam), and NANOG (ab21624, Abcam), followed by incubation with a FITC-conjugated secondary antibody.

### Lentiviral transduction of PDGF or HO-1 in AD-MSCs

Canine AD-MSCs were transduced with lentiviral vectors encoding green fluorescent protein (GFP) and canine PDGF or HO-1. Each cell line was obtained using a previously published procedure [14, 20]. Briefly, canine-specific primers for PDGF or HO-1 were inserted into a pCDH-EF1-MCS-pA-PGK-copGFP-T2A-Puro vector using EcoRI and BamHI (System Biosciences). The viral packaging mix (System Biosciences) and vectors encoding PDGF or HO-1 were transfected into HEK293T cells (Thermo Scientific) for lentivirus production. Then, virus particles expressing GFP, PDGF, or HO-1 were transduced into canine AD-MSCs using 15 μg/mL polybrene (Sigma), and puromycin (1.5 μg/mL, Thermo Scientific) was added for complete selection. Transduction efficiency was analyzed 3-5 days post-transduction based on GFP fluorescence and using quantitative reverse transcription-polymerase chain reaction (real-time PCR). Thus, AD-MSCs genetically modified to overexpress PDGF (PDGF-MSCs) or HO-1 (HO-1-MSCs) were established. The AD-MSCs used in in vitro experiments were divided into the following groups: (1) GFP-MSCs, (2) PDGF-MSCs, and (3) HO-1-MSCs

### LPS-induced inflammatory model of canine chondrocytes or synovial cells

To determine the optimal concentration of lipopolysaccharide (LPS, Sigma), canine chondrocytes or synovial cells (passage 1) were incubated with medium containing 0, 0.1, 1, or 10 μg/mL LPS for 6, 24, and 72 h. Cell proliferation, mRNA expression of inflammation-related genes, and cell morphology were evaluated using the (3-[4,5-dimethylthiazol-2-yl]-5-[3-carboxymethoxyphenyl]-2-[4-sulfophenyl]-2H-tetrazolium (MTS) assay and real-time PCR.

As a control, chondrocytes alone were incubated for the entire period. The chondrocytes were exposed to LPS for 24 h and then incubated further for 24 h and 72 h with the replacement of fresh medium as in the LPS group.

### Co-culture studies

Canine chondrocytes or synovial cells (passage 1) were seeded at the density of 1 × 10^5^ cells/well in the bottom chamber of a transwell (Corning) and cultured overnight at 37 °C. Then, the culture medium was replaced with 2 mL medium alone or medium containing 1 μg/mL LPS and incubated for 24 h. After stimulation with LPS, the medium was removed and refreshed. MSCs, PDGF-MSCs, or HO-1-MSCs (5.0 × 10^4^) were seeded on 0.4 μm pore polycarbonate membrane inserts (Corning) with 0.6 mL medium. The cells were incubated further for 24 or 72 h. As a control, chondrocytes or synovial cells were incubated for the entire period and the expression of the following genes was evaluated: genes encoding pro-inflammatory markers such as IL-6, IL-1β, and tumor necrosis factor-alpha (TNF-α), chondrogenic markers such as aggrecan, collagen type 2 (COL2α1), and SOX-9, collagen degradation markers (MMP-1, MMP-3, and MMP-13), fibrotic marker (COL1α1), aggrecan degradation factors (a disintegrin and metalloproteinase with thrombospondin motifs (ADAMTS)-4 and ADAMTS-5), pain-related gene (nerve growth factor [NGF]), or anti-degradation factors (a tissue inhibitor of metalloproteinases (TIMP)-1, TIMP-2, and FGF-2).

Next, each mesenchymal stem cell plated in the upper insert was harvested and differential signaling of anti-fibrotic factors (hepatocyte growth factor, HGF), anti-inflammatory marker (TNF-simulated gene-6, TSG-6), and pro-chondrogenic markers (FGF-2 and SOX-9) was analyzed. Each mesenchymal stem cell cultured alone (without chondrocytes or synovial cells) and harvested when it reached 90% confluence was used as the control.

### Cell proliferation assay

Cell proliferation was determined using the MTS colorimetric assay after 6, 24, and 72 h to assess the effect of LPS. Twenty microliters of the MTS solution (Bio-vision) was added to each of the 96 wells. After incubation for 2 h, the absorbance was measured at 490 nm using an Epoch Gen 5.2 type reader (Bio Tek). Cell viability was estimated using the trypan blue exclusion assay.

### Real-time PCR

Total RNA was extracted using the Qiazol lysis agent (Qiagen) according to the manufacturer’s instructions. The cDNA was synthesized using the PrimeScript II first-strand cDNA synthesis kit (Takara) and then amplified using the ABI StepOnePlus real-time PCR system (Applied Biosystems) after mixing with SYBR Premix Ex Taq (Takara) and primers. The primer sequences used are listed in Additional file 1: Table S1. The mRNA level of each gene was normalized to that of glyceraldehyde3-phosphate dehydrogenase (GAPDH) and quantified using the 2^−ΔΔCt^ method.

### Alcian blue staining

Primary canine chondrocytes (passage 1) were fixed with 4% paraformaldehyde for 30 min. The cells were then stained with 1% alcian blue stain solution (Lifeline Cell Technology) and dehydrated with 0.1 N hydrochloric acid.

### Canine OA model

Sixteen male Beagle dogs (2 years old, mean body weight = 8.4 ± 2.3 kg) were included in this study. The dogs were acclimatized to animal resource facilities for 1 week before the study. Complete physical examination, orthopedic examination of all limbs, blood work, and radiographs revealed that all dogs were physically healthy and did not have any musculoskeletal diseases.

The dogs were orotracheally intubated, and anesthesia was maintained with isoflurane in oxygen. The right CrCL was transected via medial parapatellar arthrotomy. The skin and subcutaneous tissue were dissected to expose the parapatellar medial retinaculum. Then, the joint capsule was incised. To inspect the stifle joint, the infrapatellar fat pad was drawn back and CrCL was transected using a No. 65 blade (Swann Morton). None of the dogs showed any pathological changes in their articular structure. The joint capsule, fascia, subcutaneous tissue, and skin were sutured using a routine procedure. During manipulation, cranial drawer motion and cranial tibial thrust in flexion and extension of the right stifle joint were confirmed. All dogs were orally administered 22 mg/kg cefazolin 5 days after CrCL transection.

### Preparation of MSCs

Each cell type (MSCs, PDGF-MSCs, or HO-1-MSCs, passage 3) was washed with DPBS and harvested using 0.25% trypsin-EDTA (Sigma) at 90% confluence. The cells were centrifuged at 220×*g* at 4 °C for 5 min. The supernatant was removed and the cell pellets were resuspended in 1 mL sterile 0.9% normal saline.

### Intra-articular administration

The dogs were randomly assigned into four groups and subjected to one of the four treatments (normal saline, MSCs, PDGF-MSCs, or HO-1-MSCs). Synovial fluid was aspirated to ensure the needle’s location via arthrocentesis using a 23-gauge needle into the lateral portion of the patellar ligament on the stifle joint. Then, each right stifle joint was aseptically injected with normal saline or each cell type as follows. All dogs were randomly injected 6 weeks after CrCL transection.

- Control group: 1 mL sterile 0.9% normal saline
- MSC group: 1 mL canine MSCs at the density of 2 × 10^7^ cells/mL
- PDGF-MSC group: 1 mL canine PDGF-overexpressing MSCs (PDGF-MSCs) at the density of 2 × 10^7^ cells/mL
- HO-1-MSCs group: 1 mL canine HO-1-overexpressing MSCs (HO-1-MSCs) at the density of 2 × 10^7^ cells/mL

### Post-treatment evaluation

#### Evaluation of adverse effects

The dogs underwent a physical examination and orthopedic evaluation of the appendicular and axial skeletons. The orthopedic evaluation consisted of palpation of every joint (swelling, heat, redness, or pain response), with each dog being assessed by the same orthopedic surgeon. Serum C-reactive protein (CRP) concentration was measured using a commercially available kit (V Check Canine CRP 2.0 test kit, Bionote) before injection and 3 days and 1, 2, 4, and 6 weeks after injection to monitor the adverse effects.

#### Orthopedic examination

Clinical lameness, weight-bearing, and pain response to palpation were assessed via orthopedic examination 3 days and 1, 2, 4, 6, and 10 weeks after injection using a modified scoring system (Additional file 2: Table S2) [21].

#### Radiographic evaluation

Radiographs of the right stifle joint were captured before CrCL transection and 4, 8, 12, and 16 weeks. The opacity of the infrapatellar fat pad, the thickness of the patellar ligament, and osteoarthritis score were determined by a single trained veterinarian. To assess the progression of radiographic change, a quantitative radiographic score system was used according to a previous study (Additional file 3: Table S3) [22].

#### Enzyme-linked immunosorbent assay (ELISA)

The concentration of serum matrix metallopeptidase-13 (MMP-13) was measured using commercial canine MMP-13 ELISA kits (Cusabio) according to the manufacturer’s guidelines. The animals were fasted for 12 h before blood sampling. Serum MMP-13 levels were measured 3 days and 1, 2, 4, and 6 weeks after injection.

#### Statistical analysis

Quantitative data are reported as mean ± standard deviation (SD). Statistical analysis was performed using GraphPad Prism 8.0.1. The Mann-Whitney test was used to analyze statistical differences between two groups, and one-way analysis of variance (ANOVA) with Bonferroni’s post hoc analysis was performed to determine the statistical difference among groups. Two-way ANOVA analyses with Tukey’s multiple comparison tests were performed in vitro. In vivo data were analyzed using two-way ANOVA with Dunnett’s multiple comparison tests. The results were considered statistically significant at *P* < 0.05.

## Results

### Isolation and characterization of canine AD-MSCs

**Fig. 1:**
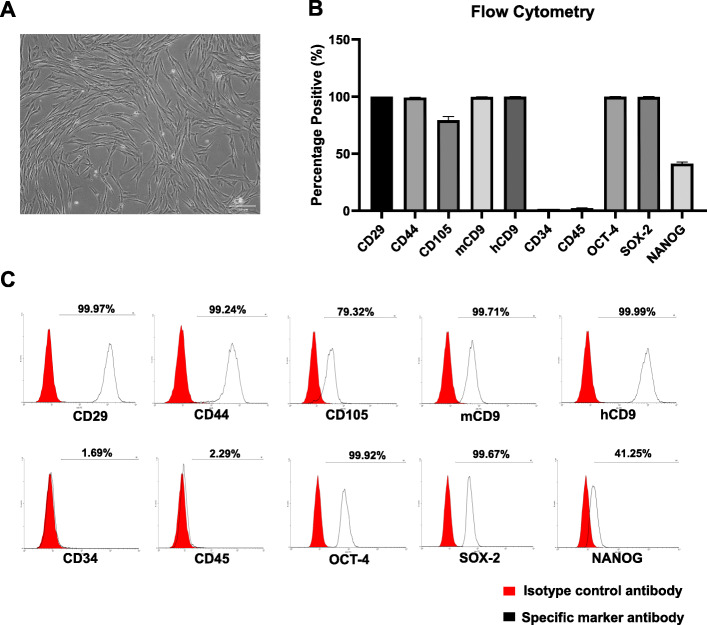
Isolation and characterization of canine AD-MSCs. **a** Representative phase-contrast microscopy image 7 days post isolation (scale bar = 200 μm). **b** Flow cytometry analysis of canine AD-MSCs at passage 1 (percentage of cells staining positive for each marker). **c** Representative histograms of flow cytometry analysis. The red histogram indicates the isotype-matched control antibody and the black one represents the specific marker staining. Data are expressed as mean ± SD

Following cell expansion, the cells showed spindle-shaped morphology (Fig. 1a) and reached 90% confluence approximately after 7 days of culture. Canine AD-MSCs showed increased expression of mesenchymal-specific markers and pluripotency markers, while the expression of these markers in hematopoietic cells decreased (Fig. 1b). Flow cytometry analyses revealed that cells at passage 1 primarily expressed the mesenchymal markers, CD29, CD44, CD105, mCD9, and hCD9 (99.97 ± 0.02%, 99.24 ± 0.12%, 79.32 ± 3.20%, 99.71 ± 0.14%, and 99.99 ± 0.00% respectively), as shown by the representative histograms (Fig. 1c). The expression of the pluripotency markers, OCT-4, SOX-2, and NANOG (99.92 ± 0.11%, 99.67 ± 0.40%, and 41.25 ± 1.48%), was also high.

### Efficient transduction of canine PDGF or HO-1 in AD-MSCs

**Fig. 2:**
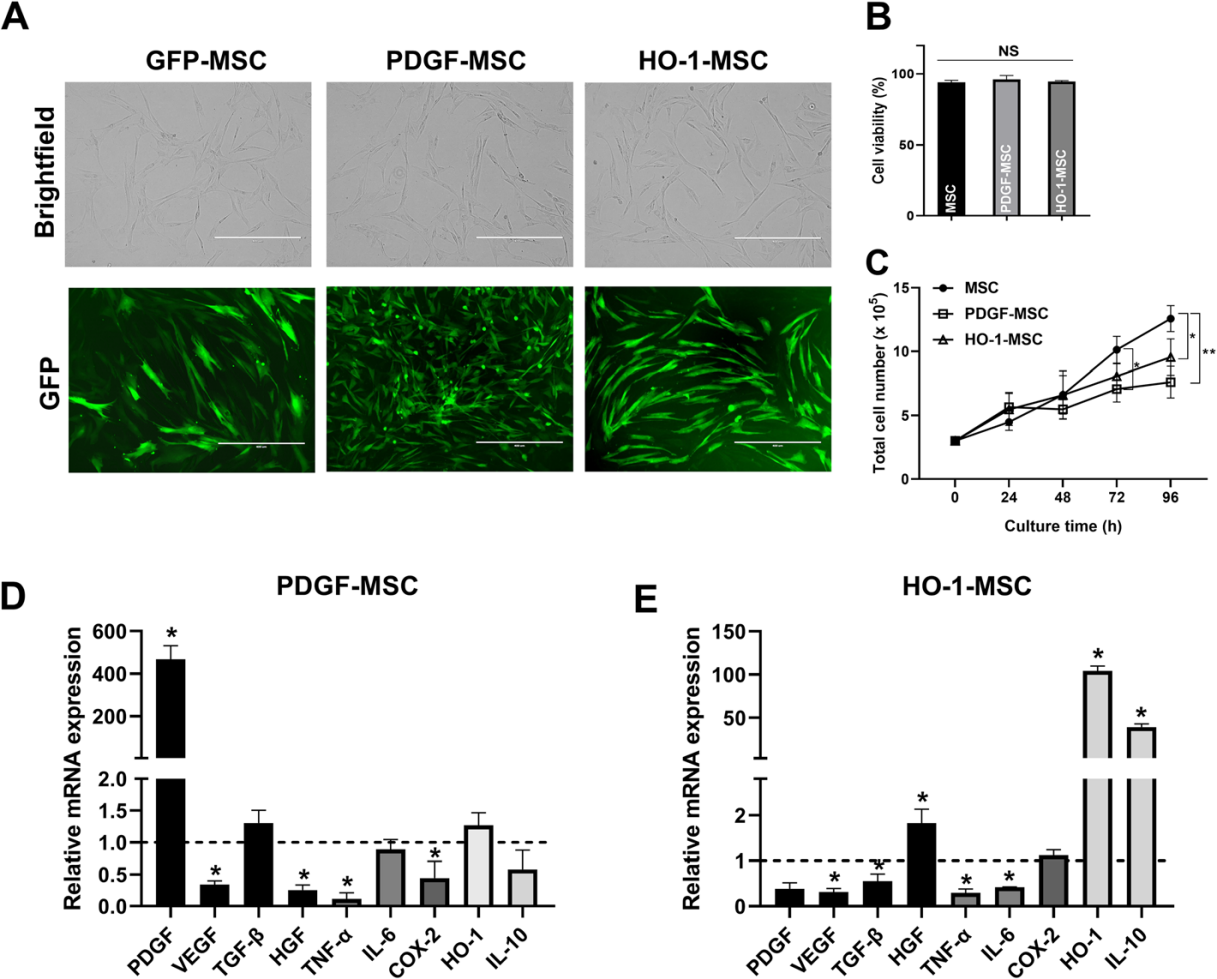
PDGF or HO-1 lentiviral transduction efficiency in canine AD-MSCs. **a** Brightfield and GFP images of canine AD-MSCs transduced with PDGF or HO1; GFP expression indicates the successful transduction of PDGF or HO1 (scale bar = 400 μm). **b** Cell viability assay at passage 3. **c** Time course of cell proliferation. **d**, **e** Relative mRNA expression of various genes in PDGF-MSCs and HO-1-MSCs. Data are expressed as fold change of gene expression compared to GFP-MSCs (dashed line). *P < 0.05 and **P < 0.01 compared with the GFP-MSC group. NS, not significant

For lentiviral transduction, the AD-MSCs were characterized using flow cytometry and transduced with commercially available lentivirus vectors expressing GFP, PDGF, or HO-1. After transduction, each cell line stably expressing the target gene was subjected to antibiotic selection. Efficient transduction of MSCs was confirmed using GFP expression (Fig. 2a), and no significant differences in cell viability were detected between groups after gene transfer (Fig. 2b). However, PDGF-MSCs demonstrated a longer doubling time than MSCs and HO-1-MSCs (Fig. 2c). In addition, we confirmed that the mRNA levels of PDGF or HO-1 were upregulated in each cell line (Fig. 2d, e). Real-time PCR also showed a significant decrease in the expression of pro-inflammatory factors such as TNF-α, IL-6, or cyclooxygenase-2 (COX-2) in both PDGF-MSCs and HO-1-MSCs. The mRNA levels of HGF and IL-10 were also significantly increased in HO-1-MSCs.

### Isolation and expansion of canine chondrocytes or synovial cells

**Fig. 3:**
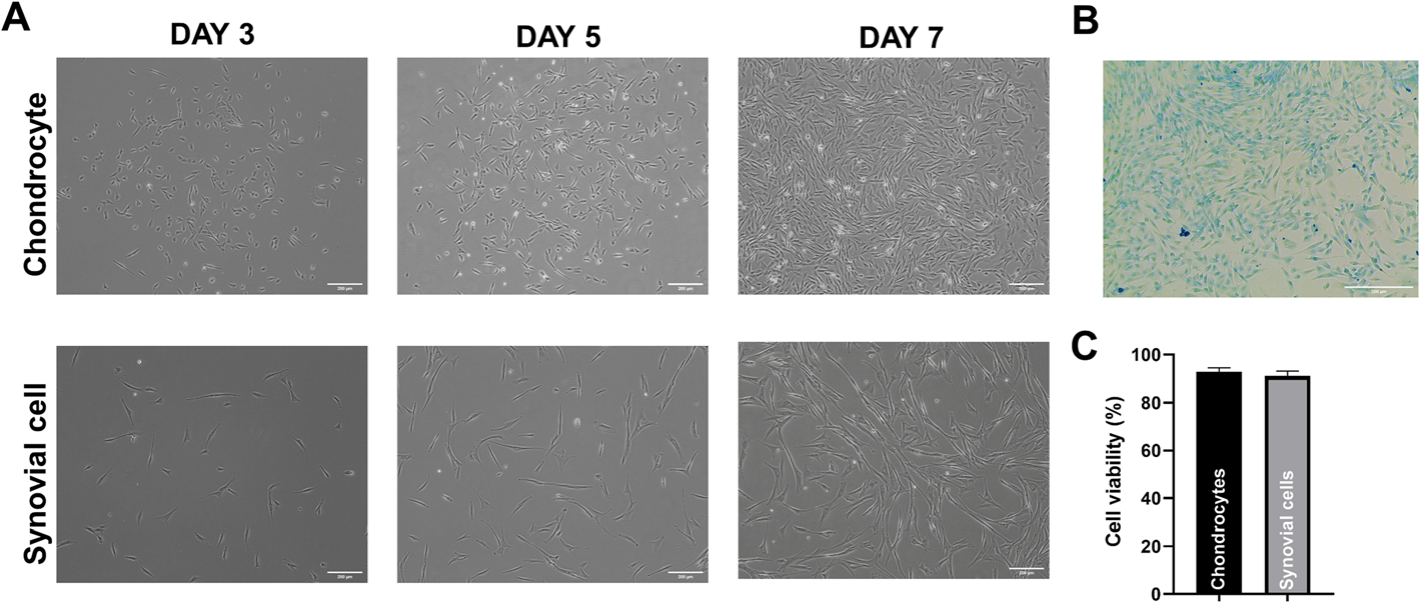
Primary culture of canine chondrocytes or synovial cells. **a** Morphology of canine chondrocyte or synovial cell cultures (passage 1) 3, 5, and 7 days after seeding (scale bar = 200 μm). **b** Alcian blue staining of canine chondrocytes (scale bar = 200 μm). **c** Cell viability assay of canine chondrocytes and synovial cells at 80% confluency (passage 1)

On day 3, canine chondrocytes were characterized by a mixture of cells with polygonal and spindle shapes (Fig. 3a). After 5 days of culture, a homogenous population of fibroblast-like cells formed a dense monolayer. Canine synovial cells were characterized by a consistent spindle shape during expansion. Alcian blue staining was used to detect proteoglycans in the extracellular matrix of chondrocytes at passage 1 (Fig. 3b). Proteoglycan was detected in all chondrocytes in the representative image. When each cell line was grown to 80% confluence at passage 1, cell viability was 92.75 ± 1.7% for chondrocytes and 91.0 ± 2.16% for synovial cells in the trypan blue assay (Fig. 3c).

### LPS induced an inflammatory response in canine chondrocytes and synovial cells

**Fig. 4:**
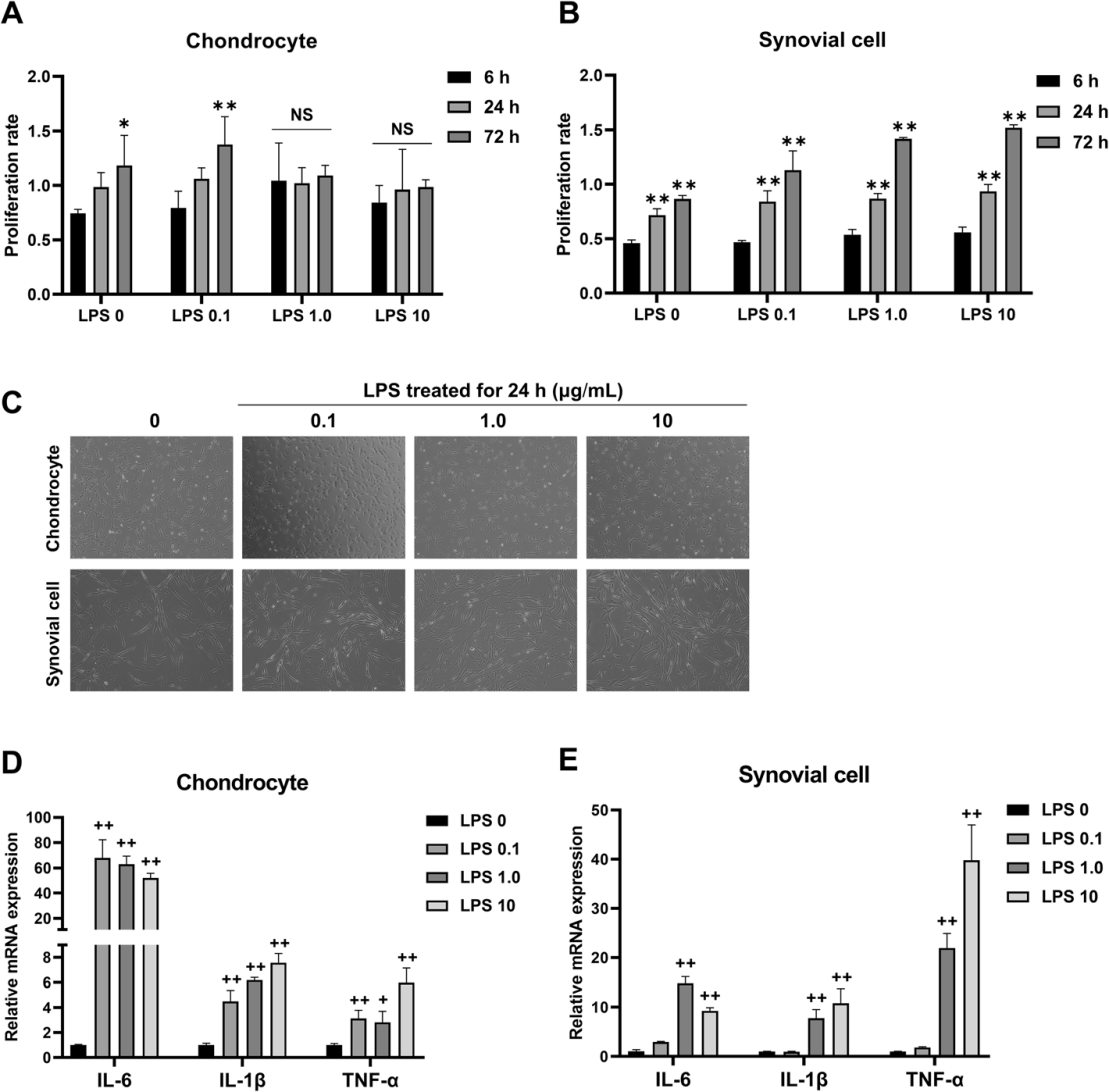
LPS-induced inflammation model of canine chondrocytes or synovial cells. **a**, **b** Cell proliferation rate of chondrocytes or synovial cells when exposed to a variable dose of LPS for 6, 24, and 72 h measured by MTS assay. *P < 0.05 and **P < 0.01 compared to the 6 h group at specific LPS concentrations. NS, not significant. **c** Representative phase-contrast images of chondrocytes or synovial cells incubated with LPS for 24 h (× 100 magnification). **d**, **e** mRNA expression of inflammation-related genes after exposure with 0 (control), 0.1, 1, and 10 μg/mL LPS for 24 h. ^**+**^*P* < 0.05 and ^**++**^*P* < 0.01 compared to the control group

To determine the optimal concentration of LPS, we first investigated the effect of LPS on cell proliferation. Chondrocytes or synovial cells were incubated with 0, 0.1, 1, and 10 μg/mL LPS for 6, 24, and 72 h and then evaluated using the MTS assay. After LPS treatment for 72 h, the proliferation rate of chondrocytes incubated with 0 and 0.1 μg/mL LPS increased significantly (Fig. 4a). However, no significant difference was observed when incubated with 1 and 10 μg/mL LPS. This showed that 1.0 and 10 μg/mL LPS was sufficient for inhibiting chondrocyte proliferation. In contrast to chondrocytes, incubation of synovial cells with 0.1-10 μg/mL LPS did not inhibit cell proliferation (Fig. 4b). Morphological changes were not observed in the LPS-treated groups compared to the control group (Fig. 4c). We next confirmed that the mRNA levels of inflammation-related genes in chondrocytes significantly increased when incubated with 0.1, 1, and 10 μg/mL LPS for 24 h (Fig. 4d). The mRNA levels of inflammatory genes in synovial cells started to increase when exposed to 1 μg/mL LPS for 24 h (Fig. 4e). Based on these results, we selected the optimal concentration of LPS as 1 μg/mL and incubation time as 24 h to establish in vitro inflammatory chondrocytes or synovial cell models.

### MSC-co-culture decreased inflammation and ECM degradation but increased proteoglycan synthesis

**Fig. 5:**
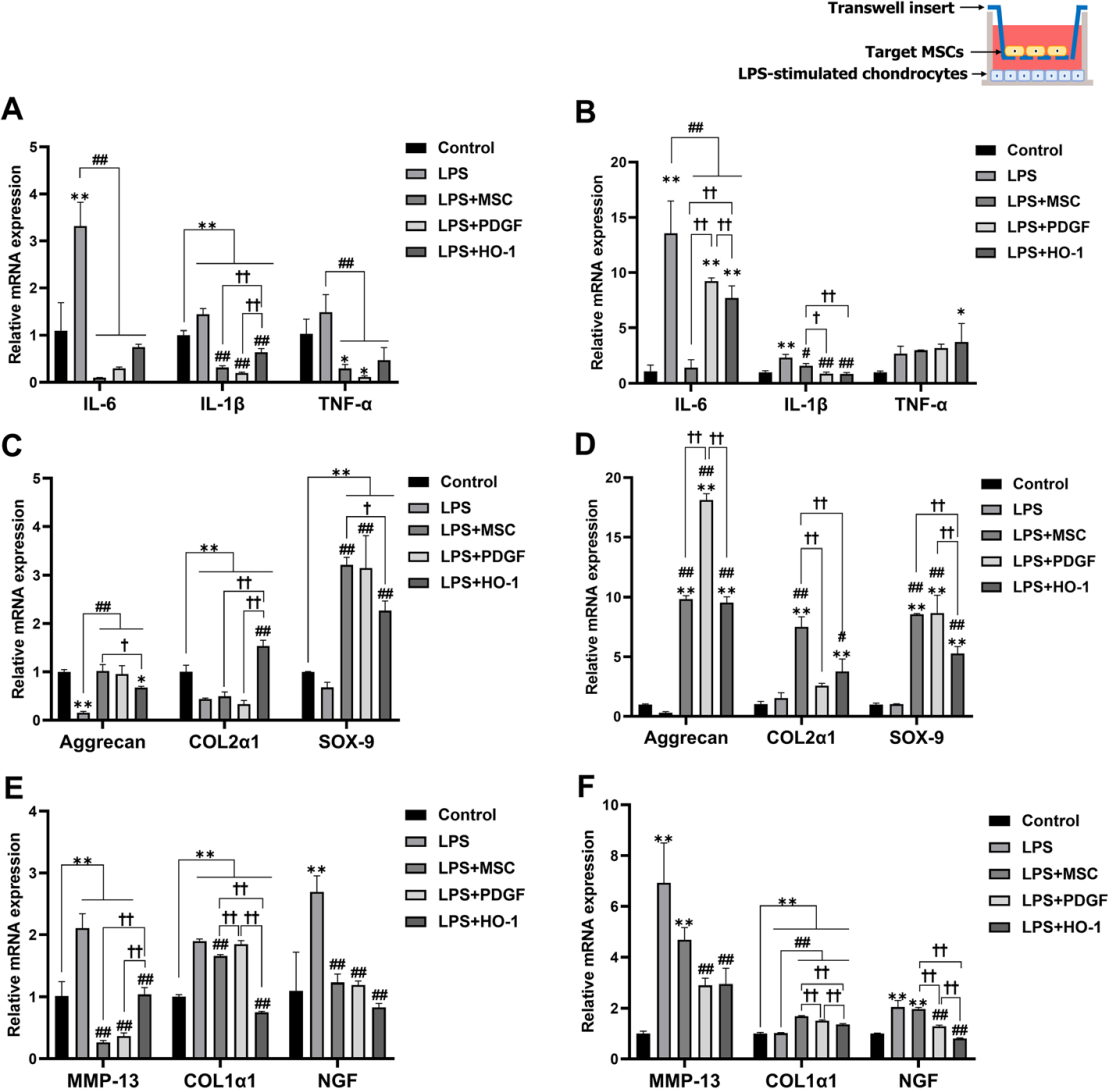
Gene expression of LPS-stimulated chondrocytes when co-cultured with MSCs, PDGF-MSCs, or HO-1-MSCs. **a**, **b** Pro-inflammatory factors after 24 and 72 h of incubation. **c**, **d** Chondrogenic marker expression. **e**, **f** Expression of hypertrophic (MMP-13), fibrotic (COL1α1), and pain-related (NGF) markers. Data are shown as fold change compared to chondrocytes alone. *P < 0.05 and **P < 0.01 compared to the control. ^#^*P* < 0.05 and ^##^*P* < 0.01 compared to the LPS group. ^†^*P* < 0.05 and ^††^*P* < 0.01, a significant difference between the indicated groups

We designed a non-contact co-culture system using a transwell in which the three different types of MSCs were seeded on the inserts after LPS exposure. The groups included only chondrocytes (Control), none (LPS), MSCs (LPS + MSC), PDGF-MSCs (LPS + PDGF), or HO-1-MSCs (LPS + HO-1). Canine chondrocytes (bottom) and target MSCs (upper part) were incubated together for 24 h (Fig. 5a, c, and e) and 72 h (Fig. 5b, d, and f) after LPS stimulation.

At 24 h, we observed that the expression of pro-inflammatory factors IL-6, IL-1β, and TNF-α in LPS-stimulated chondrocytes in all co-culture groups decreased significantly (Fig. 5a). In the LPS group, aggrecan, COL2α1, and SOX-9 expressions decreased significantly compared to those of the control (Fig. 5c). However, in the presence of MSCs, the expression of chondrogenic markers (aggrecan and SOX-9) increased significantly in all co-culture groups compared to that in the LPS group. In particular, COL2α1 expression did not change in MSCs and PDGF-MSCs, although a significant increase in COL2α1 expression was observed in HO-1-MSCs after 24 h of incubation. Similarly, the expression of the hypertrophic markers, MMP-13 and COL1α1, increased significantly in the LPS group, while MSCs and HO-1-MSCs showed significant downregulation of MMP-13 and COL1α1 (Fig. 5e). PDGF-MSCs maintained the expression of COL1α1 with no significant difference, although MMP-13 expression was significantly downregulated compared to that in the LPS group. The mRNA level of NGF, a pain-related factor, was significantly upregulated in the LPS group compared to that in the control, while all co-culture groups showed significant downregulation compared to the LPS group.

After further incubation for 72 h, the mRNA levels of IL-6 and IL-1β in all co-culture groups decreased significantly compared to those in the LPS group, while the level of TNF-α did not differ significantly (Fig. 5b). However, unlike that observed after 24 h of incubation, PDGF-MSCs and HO-1-MSCs showed a higher IL-6 mRNA level than the control group. The expression of chondrogenic markers, including aggrecan, COL2α1, and SOX-9, increased significantly in the MSCs and HO-1-MSCs co-culture compared to that in the LPS group (Fig. 5d). PDGF-MSCs also showed a significant increase in the mRNA levels of aggrecan and SOX-9, but not that of COL2α1. Regarding the hypertrophic markers, MMP-13 was significantly downregulated in PDGF-MSCs and HO-1-MSCs, compared to the LPS group after 72 h (Fig. 5f). All co-culture groups showed a significant increase in COL1α1 expression compared to that in the LPS group. The mRNA levels of NGF in MSCs, PDGF-MSCs, and HO-1-MSCs were significantly downregulated. These results demonstrated that co-culture with MSCs, PDGF-MSCs, or HO-1-MSCs inhibited the inflammatory pathway and degradation of the extracellular matrix and pain-related factors and enhanced proteoglycan synthesis via paracrine secretion.

### MSC-co-culture enhanced the contribution of synovial cells to OA recovery

**Fig. 6:**
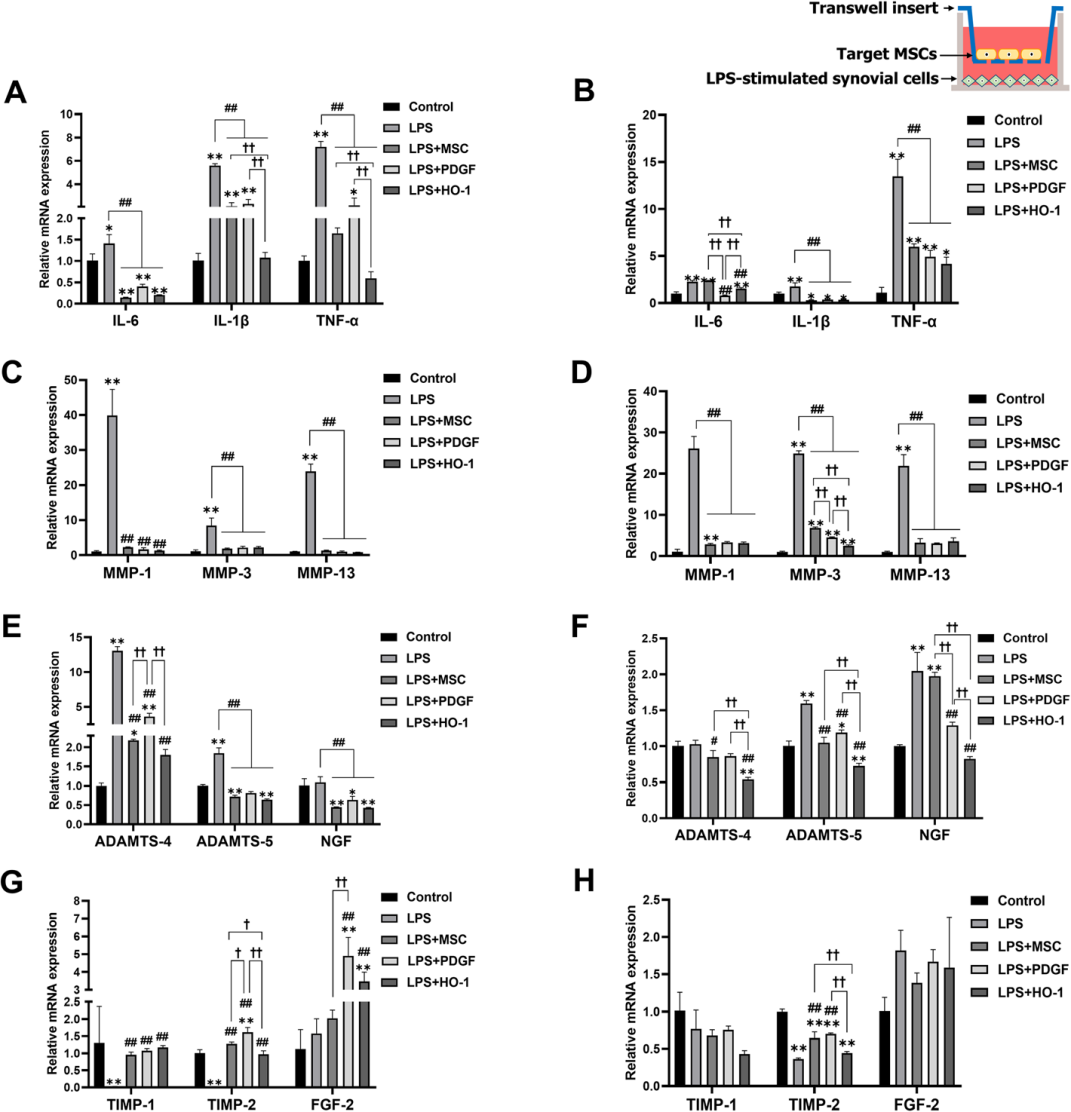
Gene expression of LPS-stimulated synovial cells when co-cultured with MSCs, PDGF-MSCs, or HO-1-MSCs. **a**, **b** Pro-inflammatory factor expression after 24 and 72 h of incubation. **c**, **d** Collagen degradation markers. **e**, **f** Expression of aggrecan degradation markers (ADAMTS-4 and ADAMTS-5) and pain-related marker (NGF). **g**, **h** Expression of anti-degradation factors. Data are shown as fold change compared to chondrocytes alone. *P < 0.05 and **P < 0.01 compared to the control. ^#^*P* < 0.05 and ^##^*P* < 0.01 compared to the LPS group. ^†^*P* < 0.05 and ^††^*P* < 0.01, a significant difference between the indicated groups

We also investigated whether genetically engineered MSCs can modulate the signals of synovial cells after LPS exposure and analyzed each co-culture group. The groups included only synovial cells (Control), none (LPS), MSCs (LPS + MSC), PDGF-MSCs (LPS + PDGF), or HO-1-MSCs (LPS + HO-1). At 24 h, pro-inflammatory factors were significantly upregulated in the LPS group compared to the control group (Fig. 6a). In contrast, the mRNA levels of all pro-inflammatory factors in the co-culture groups decreased significantly compared to those in the LPS group. Among the three co-culture groups, HO-1-MSCs showed the lowest expression of IL-1β, which was significantly different from that of the MSC and PDGF-MSC groups. Moreover, the expression of collagen degradation factors, such as members of the MMP families, in the LPS group was significantly higher than that in the control group (Fig. 6c). All co-culture groups showed significant downregulation of MMP-1, MMP-3, and MMP-13 levels. The mRNA levels of genes associated with aggrecan degradation, including ADAMTS-4 and ADAMTS-5, increased significantly in the LPS group (Fig. 6e), whereas MSCs, PDGF-MSCs, and HO-1-MSCs showed a significant decrease in the expression of these degradation markers and NGF. In particular, HO-1-MSCs showed the most significant decrease in the expression of ADAMTS-4. Furthermore, incubation with LPS alone decreased the expression of anti-degradation markers, including TIMP-1 and TIMP-2 (Fig. 6g). On the contrary, the mRNA levels of TIMP-1 and TIMP-2 were significantly upregulated in all co-culture groups. In particular, PDGF-MSCs showed the most significant increase in TIMP-2 expression. TIMP is reported to directly stop ECM proteolysis or indirectly assist in ECM synthesis. These results suggested that TIMP expression inhibited all types of MMP and that MMP expression is regulated by TIMP via negative feedback, as shown previously. FGF-2, known to enhance chondrogenesis in canine cartilages, was upregulated significantly in PDGF-MSCs and HO-1-MSCs, but not in MSCs.

After 72 more hours, a significant decrease in the level of pro-inflammatory factors and MMP families was observed in all co-culture groups (Fig. 6b, d). PDGF-MSCs showed a greater reduction in the IL-6 level. Co-culture with HO-1-MSCs showed significant downregulation of MMP-3 compared to MSCs and PDGF-MSCs. The MSC and HO-MSC groups also showed significant downregulation of ADAMTS-4 and ADAMTS-5, elicited by LPS (Fig. 6f). PDGF-MSCs and HO-1-MSCs showed a higher reduction in NGF levels than the other groups. TIMP-1 and FGF-2 expression after 72 h of incubation did not differ significantly among the groups (Fig. 6h). However, TIMP-2 mRNA level increased in the MSC and PDGF-MSC groups compared to that in the LPS group; PDGF-MSCs showed the highest upregulation of TIMP-2 among the co-culture groups.

### MSCs exerted their anti-fibrotic, anti-inflammatory, and pro-chondrogenic capacities using PDGF and HO-1

**Fig. 7:**
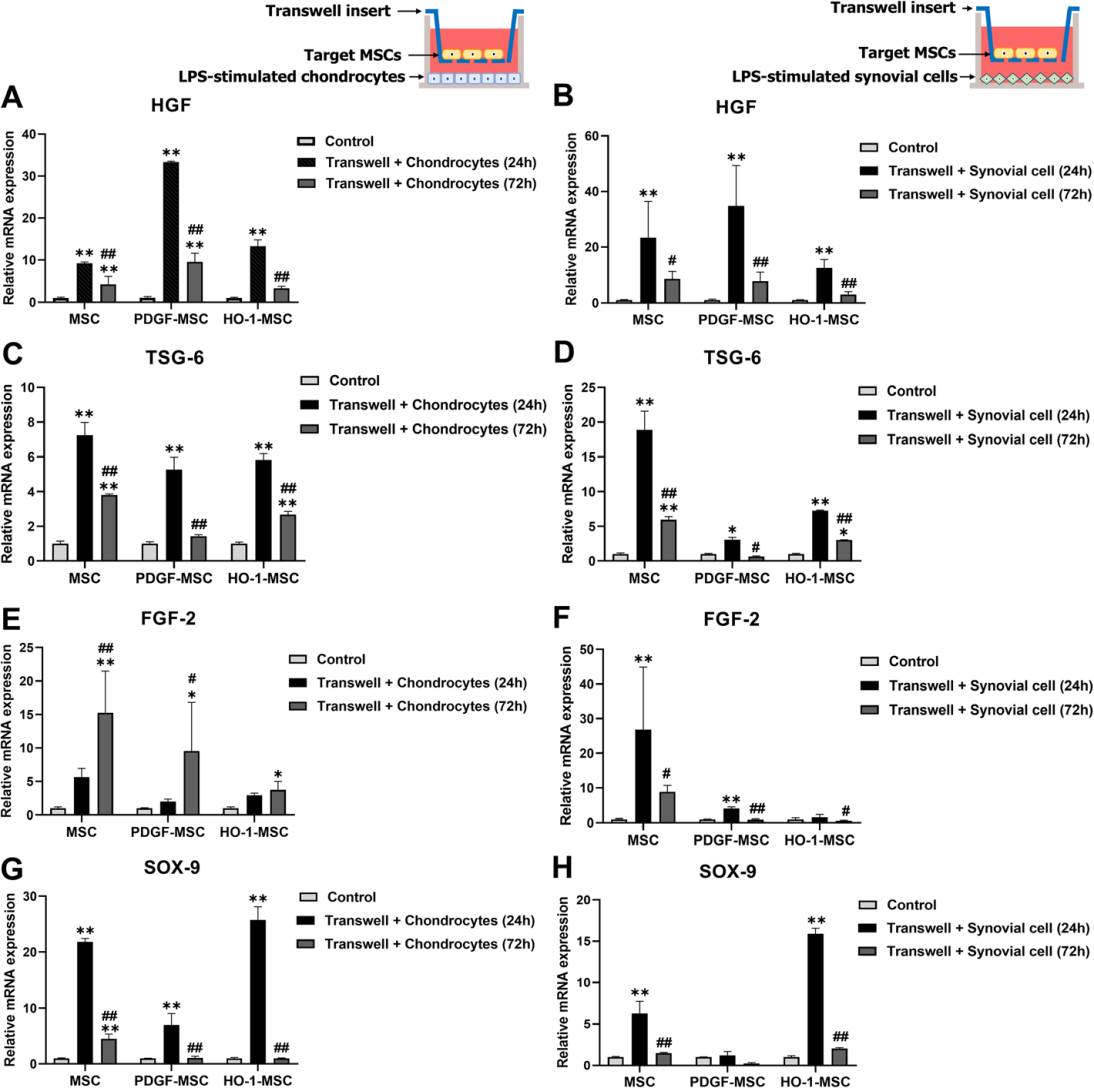
Gene expression of MSCs, PDGF-MSCs, and HO-1-MSCs after co-culture for 24 and 72 h. **a**, **b** Expression of anti-fibrotic marker HGF when co-cultured with chondrocytes and synovial cells. **c**, **d** Expression of anti-inflammatory marker TSG-6. **e–h** Expression of pro-chondrogenic markers FGF-2 and SOX-9. *P < 0.05 and **P < 0.01 compared to the control within the groups. ^#^*P* < 0.05 and ^##^*P* < 0.01 compared to the 24 h incubation within groups

We examined whether the properties of MSCs changed when co-cultured with inflamed cells or when incubated for 72 h. First, the level of HGF, an anti-fibrotic factor, increased significantly after 24 h of incubation in all the co-culture groups (Fig. 7a). On the other hand, HGF expression after 72 h was significantly lower than after 24 h in all the co-culture groups but was significantly higher in MSC and PDGF-MSC groups than in the control group. HGF expression in HO-1-MSCs did not differ significantly between the control and after 72 h of incubation. Results showed that MSCs responded to the surrounding chondrocytes in their environment. Moreover, all the co-culture groups synthesized significantly higher levels of TSG-6 mRNA after 24 h (Fig. 7c). However, this effect was lower at 72 h than at 24 h. Conversely, a significant increase in FGF-2 mRNA levels was observed at 72 h in all co-culture groups (Fig. 7e). Sox-9, which is responsible for chondrogenic commitment, was significantly upregulated at 24 h when MSCs, PDGF-MSCs, or HO-1-MSCs were co-cultured with the LPS-induced chondrocytes (Fig. 7g). Compared to that in the control, we also observed a significant increase in the SOX-9 level in MSCs after 72 h of incubation. However, the SOX-9 level in PDGF-MSCs and HO-1-MSCs decreased at 72 h. Overall, our results indicated that the production of cytokines in MSCs increased in a new environment in the early phase but was controlled when the cells were cultured for longer.

Next, we analyzed MSCs co-cultured with inflamed synovial cells. The effect of synovial cells on the anti-fibrotic activity of MSCs was assessed by measuring HGF levels, which demonstrated an enhanced production of this enzyme in MSCs surrounding the inflamed synovial cells. The HGF expression in all-co-culture groups was also higher than that in the control at 24 h (Fig. 7b). The anti-inflammatory capacity of MSCs was also evaluated based on TSG-6 expression. At 24 h, all cell populations showed a significant increase in TSG-6 expression compared to that in the control group (Fig. 7d). Furthermore, we observed that the FGF-2 level in the MSC and PDGF-MSC groups at 24 h was significantly higher than that in the control (Fig. 7f). However, FGF expression decreased at 72 h. On the other hand, HO-1-MSCs did not show any significant changes in the expression of FGF-2 at 24 h and 72 h. SOX-9 expression in MSCs and HO-1-MSCs was significantly higher than that in the control, while PDGF-MSCs maintained SOX-9 expression irrespective of co-culture with synovial cells (Fig. 7h). These results suggest that MSCs, PDGF-MSCs, or HO-1-MSCs incubated with inflamed synovial cells may regulate OA progression in terms of fibrosis, inflammation, and chondrogenesis.

### Intra-articular injection of genetically modified MSCs ameliorated OA symptoms in a canine OA model

The safety of administering MSCs, PDGF-MSCs, and HO-1-MSCs was monitored by measuring the level of serum CRP before injection and physical examination. Although one dog showed an elevated level of CRP (33.6 mg/L, normal range < 20 mg/L) 3 days post-intra-articular injection of HO-1-MSCs, adverse clinical effects were not observed. After 4 days, the CRP level returned to the normal range (< 10 mg/L).

**Fig. 8:**
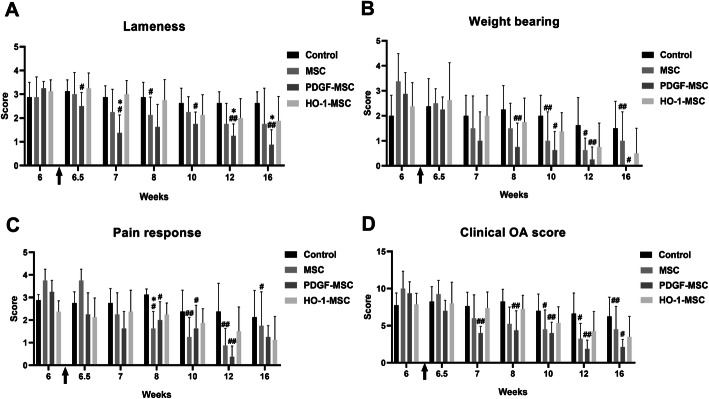
Assessment of OA symptoms, including lameness, weight-bearing, pain response, and clinical OA scores. **a** Lameness score in the affected knees of dogs. **b** Weight-bearing score. **c** Severity of pain response. **d** Clinical OA scores. The arrow indicates the time point of intra-articular injection (6 weeks) of normal saline (Control) or target cells. *P < 0.05 and **P < 0.01 compared to the control at specific time points. ^#^*P* < 0.05 and ^##^*P* < 0.01 compared to before treatment within groups

Pretreatment mean lameness scores did not differ significantly between groups (Fig. 8a). First, in the group treated with PDGF-MSCs, the lameness scores decreased significantly 3 days after treatment (6.5 weeks) compared to those before treatment. The MSC-treated group showed a significant decrease in lameness scores at 2 weeks after treatment (8 weeks). In contrast, lameness remained unchanged over time in the control group (normal saline). Second, 2 weeks after the intra-articular injection of PDGF-MSCs (8 weeks), the weight-bearing scores were significantly lower and the reduction persisted for 4, 6, and 10 weeks after PDGF-MSC injection (Fig. 8b). In dogs treated with MSCs, weight-bearing improved significantly from 4 weeks after treatment (10 weeks) and the pain response decreased significantly 2 weeks after the intra-articular injection of MSCs (Fig. 8c). The PDGF-MSC-treated group also showed a reduction in pain scores at 2, 4, and 6 weeks after treatment.

Taken together, clinical OA scores indicated that the administration of MSCs improved the symptoms from 4 weeks after injection and that the administration of PDGF-MSCs started to ameliorate the symptoms from 1 week after injection (Fig. 8d). Regarding functional assessments over time, statistically significant improvements in lameness, weight-bearing, pain, and clinical OA scores were noted for PDGF-MSC treatment over the study period. HO-1-MSCs tended to improve the orthopedic symptoms, including lameness, weight-bearing, and pain response over time, although the difference was not statistically significant.

### Intra-articular injection of MSCs did not affect the radiographic changes or the serum MMP-13 concentration

**Fig. 9:**
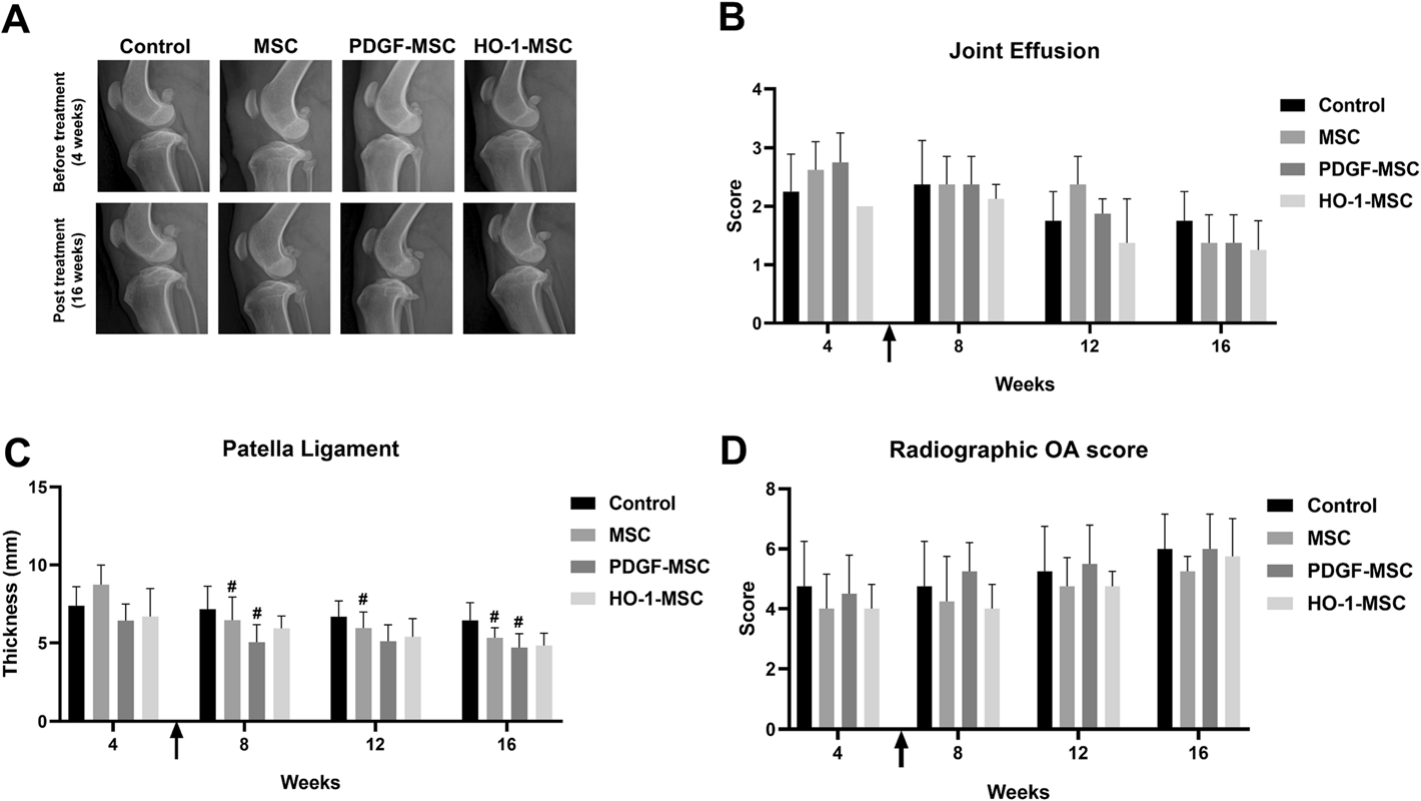
Radiographic evaluation before and after intra-articular injections. **a** Radiographic images of the affected knee of dogs before and post-treatment. **b** Joint effusion degree. **c** Thickness of the patella ligament. **d** Radiographic OA scores. The arrow shows the time point of intra-articular injection (6 weeks) of normal saline (Control) or target cells. *P < 0.05 and **P < 0.01 compared to the control at specific time points. ^#^*P* < 0.05 and ^##^*P* < 0.01 compared to before treatment within groups

We did not detect any significant difference in joint effusion. Although swelling of the patellar ligament decreased over time after treatment, the radiographic OA severity score did not differ significantly (Fig. 9). In addition, a decrease in the serum MMP-13 level was observed in all MSC-containing groups to some extent, although it did not differ significantly compared to that before treatment (Additional file 4: Table S4).

## Discussion

Once the joint fails to maintain homeostasis due to insufficiency of the cartilage repair, pro-inflammatory mediators play pivotal roles in disrupting the balance of catabolic and anabolic pathways. IL-1b, IL-6, and TNF-α play vital roles in the progression of OA. Several cell types in the joint, including chondrocytes, synovial cells, osteoblasts, and adipocytes, start to produce pro-inflammatory factors spontaneously, which strongly induce the release of proteolytic enzymes, such as MMPs and ADAMTSs. These lead to fragmentation and degradation of the ECM and work synergistically with pro-inflammatory cytokines. Considering this complexity of OA pathogenesis, we aimed to inhibit inflammation using PDGF, while promoting chondroprotection using HO-1.

Despite extensive investigations over the last decade, a complete cure for OA has not been obtained yet for humans or animals. Although many studies have reported that naïve MSCs exert positive effects on osteoarthritic cells in vitro owing to their pluripotency [23–26], in vivo studies have produced ambiguous results. Hence, in this study, we established stable cell lines of genetically engineered MSCs expressing PDGF or HO-1 via gene transfer and demonstrated that they act as sensitive and comprehensive bioagents for OA treatment. Interestingly, canine AD-MSCs transduced with PDGF or HO1 via a lentiviral vector showed high transduction efficiency and steadily overexpressed target genes in the long term. Furthermore, PDGF overexpression decreased the expression of TNF-α and COX-2. HO-1 overexpression also enhanced the expression of HGF and IL-10, while decreasing the expression of TGF-β, TNF-α, and IL-6, compared to that in naïve MSCs (Fig. 2d, e). This indicated that these modified MSCs possibly exerted strong immunomodulatory effects within the damaged joint.

In vitro results showed that synovial cells exhibited differential signaling of pro-chondrogenic and ECM-degradative factors when co-cultured with genetically modified MSCs. Maumus et al. [24] observed that MSCs exerted anti-inflammatory effects on OA chondrocytes and synovial cells. Consistent with this report, we observed downregulation of the major anti-inflammatory factors in the indirect MSC/synovial cell co-culture. Furthermore, we sought to expand the existing molecular knowledge by investigating the factors involved in OA pathogenesis. Our results showed a downregulation of MMPs and ADAMTSs, but upregulation of TIMPs and FGF-2 (Fig. 6), indicating that synovial cells are important components for changing the microenvironment and contribute to the progression of OA within the inflamed joint. In addition, all MSC groups accelerated the expression of HGF, TSG-6, FGF-2, and SOX-9 when they met the damaged chondrocytes or synovial cells (Fig. 7), suggesting that genetically engineered MSCs empower both chondrocytes and synovial cells to control inflammation and protect them from the ECM-degradative microenvironment.

Next, we investigated whether the duration of co-culture with PDGF-MSCs or HO-1-MSCs affected the signaling pattern of chondrocytes or synovial cells. The alteration in the expression of several markers, including MMPs, aggrecan, and SOX-9, was increased in PDGF-MSCs or HO-1-MSCs after 72 h of incubation. However, unexpectedly, the mRNA level of IL-6 was relatively high at 72 h in LPS-stimulated chondrocytes. In addition, the mRNA level of COL1α1 decreased significantly in all MSC groups at 24 h, although this effect was reversed at 72 h. The possible reason for this reversion of results is the decrease in the cell proliferation rate. As shown in Fig. 2c, both PDGF-MSCs and HO-1-MSCs require a longer time to duplicate than MSCs. Therefore, taking cell numbers into account, the strong downregulation of IL-6 mRNA at the early phase might be reversed at 72 h.

It is well known that MSCs exert strong immunomodulatory effects when primed with inflammatory cytokines in humans and mice [27, 28]. However, the immunomodulatory effects of canine MSCs on chondrocytes or synovial cells have not been elucidated yet. Indeed, our study showed that the expression of soluble factors such as HGF, TSG-6, FGF-2, and SOX-9 increased when co-cultured with damaged chondrocytes or synovial cells. Among them, HGF has been identified to be crucial for the protective effects of MSCs [29]. Interestingly, we observed that PDGF-MSCs significantly enhanced the expression of HGF during co-culture with both damaged chondrocytes and synovial cells. This suggested that PDGF-MSCs play a potent cytoprotective role in experimentally induced osteoarthritis, preventing chondrocyte apoptosis and ECM degradation, and improving impaired cell homeostasis.

Using canine OA models, we sought to determine whether genetically engineered MSCs could safely improve OA symptoms. No major complications due to the intra-articular injection of cells were observed during the entire period of the study. We administered intra-articular injections of the suspension of target cells at 6 weeks after CrCL transection. Previously, Gouze et al. had demonstrated that the expression of the IL-1 receptor antagonist via lentiviral transduction persisted for 20 days in immunocompetent animals, but at least 6 weeks in immunocompromised rats [30]. These results suggested that lentiviral vectors can deliver transgenes to the synovium efficiently and provide long-term expression under a less immune-reactive environment. Furthermore, Kondo et al. showed that an inflammatory environment strongly inhibits the differentiation of MSCs into chondrocytes, which results in the formation of calcifications [23]. Based on these studies, we administered intra-articular injections of MSCs at 6 weeks post-surgery.

We also observed significant improvements in OA clinical signs after treatment. PDGF-MSCs induced an improved reduction of OA symptoms; however, HO-1-MSCs did not produce effects significantly different from the control. HO-1 is a stress-responsive enzyme that is rapidly induced by free radicals and hypoxia [16]. Furthermore, HO-1 has been reported as a major modulator of acute inflammatory conditions in vitro and in vivo models of acute inflammation and organ diseases [31]. Although HO-1-MSCs contributed more to the regulation of cell signals in vitro, the canine OA model in this study closely resembled a chronic case rather than an acute case, as the HO-1-MSCs were applied 6 weeks post-surgery. Thus, these conditions are not optimal for HO-1-MSCs, as the stimulus for HO-1 had already disappeared. Although many previous studies have reported the efficacy of intra-articular injections of MSCs for treating OA, the duration of the therapeutic effects of genetically modified MSCs in dogs remained unclear. We observed that MSCs exerted beneficial effects for at least 6 weeks following injection when analyzing the clinical signs.

According to radiographic evaluation, no remarkable improvement was observed in any of the groups. These results are in agreement with those of previous studies that reported that radiographic OA severity did not correlate with limb function [32, 33]. In addition, we did not observe any significant differences in serum MMP-13 concentration before and after treatment. Ozler et al. [34] showed that synovial MMP-13, but not serum MMP-13 levels increased with OA progression, with a significant association with pain scores. Ideally, collection of the synovial fluid from canine OA models at each time point is preferable, although this method is limited by the lack of repeatability owing to the limited synovial fluid volume from a single dog.

This study had several limitations. First, our canine surgical OA models could not represent all types of OA. Surgery induction by CrCL transection brought advantages, including a rapid onset of OA similar to humans and the control of subject age, sex, and breed [35]. However, there are significant pathological differences between surgery-induced and naturally occurring OA in the affected joint. Liu et al. [36] found that proteoglycan levels on the articular cartilages of a surgery-induced OA model were significantly higher than those of dogs with spontaneous OA, which showed that rapid onset by surgery promoted a more active repair activity. Furthermore, OA can develop in dogs at an early age depending on genetic predisposition, and the progression and symptoms vary widely between dogs. In our study, we used only male, 2-year-old, beagle dogs, which was the optimal setting to evaluate the efficacy of the genetically modified MSCs. Based on these findings, it is necessary to conduct a clinical study in client-owned dogs suffering from naturally occurring OA for further understanding of MSC-based cell therapies. Second, we did not employ an objective numerical rating scale like force plate analysis. Recently, force plate analysis has been used to evaluate the weight-bearing/strength and range of motion of each joint and gait pattern. Powerful tools as this can assess clinical OA symptoms objectively, but a subjective evaluation works well in cases of acute and chronic limping involving the knee joint in dogs [37].

In this study, we first investigated whether genetic engineering of canine AD-MSCs with the PDGF or HO-1 gene could modulate the catabolic and anabolic activities in the inflamed joint. Based on the results of our in vitro and in vivo experiments, we concluded that PDGF-MSCs contributed the most to OA treatment modulation. We believe that PDGF-MSCs possess the advantages of both MSCs and PRP, thus recruiting endogenous stem/progenitor cells to injured tissues and stimulating the chondrogenic potential and anti-inflammatory characteristics of MSCs by overexpression of PDGF. Thus, we identified the potential of genetically engineered MSCs for clinical use. Gene transfer provides a way of overcoming the problem associated with the delivery of bioagents to the interior of the joint. Although gene therapy for OA has been discussed for decades, its clinical application has been insufficient. Similar to cell-based therapies, genetically engineered MSCs can exert synergistic effects in combination with hyaluronic acid, scaffolds, specific exosomes, or disease-modifying OA drugs [38]. In this study, we have shown the efficacy of these processes in dogs; moreover, our method can be easily adapted to study target joint inflammation in the future.

## Conclusions

Considering the complexity of OA pathogenesis, we speculated that, compared to naïve MSCs, inflammation inhibition by PDGF, along with the promotion of chondroprotection using HO-1, might improve joint preservation. Genetically engineered MSCs exerted their immunomodulatory effects on OA chondrocytes and synovial cells by upregulating anti-fibrotic and pro-chondrogenic factors. Furthermore, intra-articular injection of gene-modified MSCs ameliorated the clinical symptoms of OA in canine OA models. In particular, PDGF-MSCs acted as a superior alternative to substantially improve patient outcomes. These findings may be potentially significant for the application of cell-based therapies in OA treatment.

## Supplementary Information

**Additional file 1: Table S1.** Primers used for real-time PCR analysis.

**Additional file 2: Table S2.** The criteria of orthopedic examinations.

**Additional file 3: Table S3.** The radiographic OA score of stifle joint.

**Additional file 4: Table S4.** Mean ± SD of the serum MMP-13 concentration before and after intra-articular administration.

## Data Availability

The datasets used and/or analyzed during the current study are available from the corresponding author on reasonable request.

## Abbreviations

ADAMS: A disintegrin and metalloproteinase with thrombospondin motifs
CrCL: Cranial cruciate ligament
HGF: Hepatocyte growth factor
LPS: Lipopolysaccharide
PDGF: Platelet-derived growth factor
HO-1: Heme oxygenase-1
MSCs: Mesenchymal stem cells
SOX-9: Sex-determining region Y-box 9
IGF-1: Insulin-like growth factor-1
FGF: Fibroblast growth factor
TIMP: Tissue inhibitor of metalloproteinases
NGF: Nerve growth factor
OA: Osteoarthritis
