# Supplementary material for: Mesenchymal stem cells genetically engineered to express platelet-derived growth factor and heme oxygenase-1 ameliorate osteoarthritis in a canine model
Source: J Orthop Surg Res. 2021 Jan 11;16:43. doi: 10.1186/s13018-020-02178-4 (PMC7802278; doi:10.1186/s13018-020-02178-4)
Supplement: Supplementary file 1 — Additional file 1: Table S1. Primers used for real-time PCR analysis. [file 13018_2020_2178_MOESM1_ESM.pdf]

Additional file 1: Table S1 Primers used for qRT-PCR analysis

Target genes	Forward (5′–3′)	Reverse (5′–3′)
GAPDH	CATTGCCCTCAATGACCACT	TCCTTGGAGGCCATGTAGAC
IL-6	TTTTCTGCCAGTGCCTCTTT	GGCTACTGCTTTCCCTACCC
IL-1β	AGTTGCAAGTCTCCCACCAG	TATCCGCATCTGTTTTGCAG
TNF-α	AGCGCTGAGATCAATCTGCC	TCCAGCCCTGAGCCCTTAAT
COX-2	ACCCGCCATTATCCTAATCC	TCGGAGTTCTCCTGGCTTTA
Aggrecan	ACTGCTCCAGGCGTGTGATG	GACCATGTCGTGCAGGTGAC
Col1α1	ATCAGCCCTGGAGCATTTACA	AACTCTTCAGGGGATTGGTG
COL2α1	TACGGTGATGACAACCTGGC	GTTTCGTGCAGCCATCCTTC
SOX-9	AGTACCCGCACCTGCACAA	AAATGTCGCTCTCGGTGG
MMP-1	TGGAGCACTTCATAGCCAGC	GAAATGGTGACGCCATGCAG
MMP-3	ACCCCACTTTGTGGTTCGAC	ACCGGCTTGTACCTCAGTTC
MMP-13	CAGCGATGGTGATGATGATCTG	AGGGACCCCACATCTTGGT
TIMP-1	CTCACCAGAGAACCCACCAT	CCGGTTTAAGTCGGTCTGGT
TIMP-2	TCCCTGGACATCGGAGGAAA	CATCCAGAGGCACTCATCCG
ADAMTS-4	CTCGGACCCTGACCACTTTG	GAGCATGTTGAAGACGTGGC
ADAMTS-5	CACCCAGAAACAATGGTCGC	ACCACGTAGTAACCAGTGCC
NGF	CTTCGTCCCACCCTGTCTTC	AGTTCCAGTGCTTGGAGTCG
PDGF-B	TGTCTCTCTGCTGCTACCTG	CCAGAATGGGATCGGGTCAA
VEGF	CTATGGCAGGAGGAGAGCAC	GCTGCAGGAAACTCATCTCC
TGF-β	CTCAGTGCCCACTGTTCCTG	TCCGTGGAGCTGAAGCAGTA
FGF-2	TGCCTACCTAGATGCTGGACA	GAGCTTTGGCCGTATTTCCATTC
HGF	ATGGGGAATGAGAAATGCAG	GACAAAAATGCCAGGACGAT
TSG-6	AGCCTGCTTGGCTGACTATG	ATTTGGAAACCTCCCGCTGT
HO-1	GCGTCGACTTCTTCACCTTC	GGTCCTCAGTGTCCTTGCTC
