# Supplementary material for: Mesenchymal stem cells genetically engineered to express platelet-derived growth factor and heme oxygenase-1 ameliorate osteoarthritis in a canine model
Source: J Orthop Surg Res. 2021 Jan 11;16:43. doi: 10.1186/s13018-020-02178-4 (PMC7802278; doi:10.1186/s13018-020-02178-4)
Supplement: Supplementary file 2 — Additional file 2: Table S2. The criteria of orthopedic examinations. [file 13018_2020_2178_MOESM2_ESM.pdf]

Additional file 2: Table S2 The criteria of orthopaedic examinations

Score criteria
Lameness score
0. Normal stance and normal lameness
1. Normal stance, but lameness after exercise
2. Normal stance, but slight lameness when walking
3. Normal or abnormal stance, but moderate lameness when walking
4. Abnormal stance and severe lameness when walking
5. Reluctant to rise, stand and walk
Weight bearing score
0. Full weight-bearing
1. Partial weight-bearing
2. Slight weight-bearing
3. Toe-touch weight bearing
4. Intermittent weight-bearing
5. Non weight-bearing
Pain score on palpation
0. No response
1. Tension and spasm of partial thigh muscles
2. Mild tension and spasm of entire thigh muscles and dog turns head in recognition
3. Moderate tension and spasm of entire thigh muscles and dog pulls limb away
4. Severe tension and spasm of entire thigh muscles and dog vocalize or become aggressive
