# Supplementary material for: Mesenchymal stem cells genetically engineered to express platelet-derived growth factor and heme oxygenase-1 ameliorate osteoarthritis in a canine model
Source: J Orthop Surg Res. 2021 Jan 11;16:43. doi: 10.1186/s13018-020-02178-4 (PMC7802278; doi:10.1186/s13018-020-02178-4)
Supplement: Supplementary file 3 — Additional file 3: Table S3. The radiographic OA score of stifle joint. [file 13018_2020_2178_MOESM3_ESM.pdf]

Additional file 3: Table S3 The radiographic OA score of stifle joint

Score : normal - 0, mild - 1, moderate - 2, severe - 3
Factor<br>1. Osteophyte formation at the proximal / distal edge of the patella<br>2. Subchondral sclerosis of the trochlear groove<br>3. Osteophyte formation on the fabella<br>4. Osteophyte formation in the long digital extensor muscle groove<br>5. Subchondral sclerosis of the tibial plateau<br>6. Osteophyte formation at the tibial attachment site of the cranial cruciate ligament<br>7. Osteophyte formation at the lateral and medial femoral condyle
