# Supplementary material for: Mesenchymal stem cells genetically engineered to express platelet-derived growth factor and heme oxygenase-1 ameliorate osteoarthritis in a canine model
Source: J Orthop Surg Res. 2021 Jan 11;16:43. doi: 10.1186/s13018-020-02178-4 (PMC7802278; doi:10.1186/s13018-020-02178-4)
Supplement: Supplementary file 4 — Additional file 4: Table S4. Mean ± SD of the serum MMP-13 concentration before and after intra-articular administration. [file 13018_2020_2178_MOESM4_ESM.pdf]

Additional file 4: Table S4 Mean ± SD of the serum MMP-13 concentration before and after intra-articular administration.

Group	Before treat	3 days	1 week	2 weeks	4 weeks	10 weeks
Control	2.272±0.81	2.007±0.37	2.043±0.49	1.955±0.31	2.091±0.47	1.886±0.22
MSCs	3.452±0.91	2.946±0.95	2.703±0.18	2.559±0.47	2.495±0.46	2.654±0.67
PDGF-MSCs	2.141±0.39	2.065±0.38	2.061±0.41	2.118±0.37	2.003±0.33	2.043±0.35
HO-1-MSCs	3.386±0.21	3.314±0.11	3.367±0.12	3.284±0.11	3.277±0.07	3.221±0.10
